# Intelligent virtual case learning system based on real medical records and natural language processing

**DOI:** 10.1186/s12911-022-01797-7

**Published:** 2022-03-04

**Authors:** Mengying Wang, Zhen Sun, Mo Jia, Yan Wang, Heng Wang, Xingxing Zhu, Lianzhong Chen, Hong Ji

**Affiliations:** 1 Information Management and Big Data Center, Peking University Third Hospital, Beijing, China; 2 Education Section, Peking University Third Hospital, Beijing, China; 3 Goodwill Hessian Health Technology Co. Ltd, Beijing, China

**Keywords:** Architectures for educational technology system, Clinical thinking ability, Virtual medical records, Distance education and online learning, Artificial intelligence

## Abstract

**Background:**

Modernizing medical education by using artificial intelligence and other new technologies to improve the clinical thinking ability of medical students is an important research topic in recent years. Prominent medical universities are actively conducting research and exploration in this area. In particular, given the shortage of human resources, the need to maintain social distancing to prevent the spread of the epidemics, and the increase in the cost of medical education, it is critical to harness online learning to promote medical education. A virtual case learning system that uses natural language processing technology to process and present a hospital’s real medical records and evaluate student responses can effectively improve medical students’ clinical thinking abilities.

**Objective:**

The purpose of this study is to develop a virtual case system, AIteach, based on actual complete hospital medical records and natural language processing technology, and achieve clinical thinking ability improvement through a contactless, self-service, trial-and-error system application.

**Methods:**

Case extraction is performed on a hospital’s case data center and the best-matching cases are produced through natural language processing, word segmentation, synonym conversion, and sorting. A standard clinical questioning data module, virtual case data module, and student learning difficulty module are established to achieve simulation. Students can view the objective examination and inspection data of actual cases, including details of the consultation and physical examination, and automatically provide their learning response via a multi-dimensional evaluation system. In order to assess the changes in students’ clinical thinking after using AIteach, 15 medical graduate students were subjected to two simulation tests before and after learning through the virtual case system. The tests, which included the full-process case examination of cases having the same difficulty level, examined core clinical thinking test points such as consultation, physical examination, and disposal, and generated multi-dimensional evaluation indicators (rigor, logic, system, agility, and knowledge expansion). Thus, a complete and credible evaluation system is developed.

**Results:**

The AIteach system used an internal and external double-cycle learning model. Students collect case information through online inquiries, physical examinations, and other means, analyze the information for feedback verification, and generate their detailed multi-dimensional clinical thinking after learning. The feedback report can be evaluated and its knowledge gaps analyzed. Such learning based on real cases is in line with traditional methods of disease diagnosis and treatment, and addresses the practical difficulties in reflecting actual disease progression while keeping pace with recent research. Test results regarding short-term learning showed that the average score (*P* < 0.01) increased from 69.87 to 85.6, the five indicators of clinical thinking evaluation improved, and there was obvious logical improvement, reaching 47%.

**Conclusion:**

By combining real cases and natural language processing technology, AIteach can provide medical students (including undergraduates and postgraduates) with an online learning tool for clinical thinking training. Virtual case learning helps students to cultivate clinical thinking abilities even in the absence of clinical tutor, such as during pandemics or natural disasters.

## Introduction

Clinical thinking ability is a critically important skill for medical professionals [1]. Clinical thinking is the basis for solving clinical problems and the bridge between theory and practice; it is the basic skill required of clinicians, the basis of clinical research, and the key aim of clinical teaching [2]. Owing to the COVID-19 pandemic and the need for social distancing, physical classes in several medical universities have been temporarily suspended, so medical students have been unable to enter the clinical ward to study [3, 4]. The limitation of human resources and the increase of educational costs have also highlighted the need for the development of a sustainable system for medical student education [5]. There is an urgent need to produce new clinical thinking training and teaching methods, which do not need students to enter clinical wards to conduct and do not require the direct supervision of senior doctors [4, 6]. Such contactless self-service learning methods can be achieved by means of simulation teaching. A virtual patient system (VPS) is a computer program that can simulate real-life clinical scenarios [7, 8]. Previous studies mainly focused on the field of virtual surgery [9], laparoscopy [10], radius fracture surgery [11], virtual ultrasound simulation training [12], and other practical teaching. However, these studies primarily relied on manual compilation of cases for virtual learning. For example, Dafli et al. [13] has designed a database containing 59 cases based on the OpenLabyrinth open-source platform. Medical teachers can add nodes, links, and virtual characters to the case database through the platform’s user interface and visual editor. Furlan, Raffaello et al. [14] developed the Hepius system to conduct virtual case teaching. Here, cases are compiled by doctors, but the functional design only allows simple medical history questions and answers, which do not identify the difficulty of a case. This system can only cover the learning needs of undergraduates. Douglass et al. [15] used a virtual patient interaction system to manually design ten cases to improve the chronic disease management skills of pharmacy students. Yoon et al. [16] instructed doctors to use Excel to compile virtual cases. This kind of manual case preparation design is simple and easy to use. It only applies to the consultation process, lacks objective inspection support, and does not conform to actual case diagnosis, treatment conditions, and disease spectrum. Unfortunately, it is difficult to manually compile cases in a manner that reflects actual disease progression.

The current study uses Natural Language Processing (NLP) to generate virtual cases from real medical records, which are then reviewed by experts for verification of the content and quality of the cases. The medical records include all the scenarios encountered during the case progression, such as medical examinations and follow-up tests. Virtual cases have been constructed for 400 real cases, covering 65 diseases in the four major departments of internal medicine, surgery, gynecology, and pediatrics. Cases are classified as per their difficulty and students are assigned varying learning levels, so as to cover the learning needs of students at various levels studying multiple specializations.

## Methods

### Database of virtual cases

**Fig. 1:**
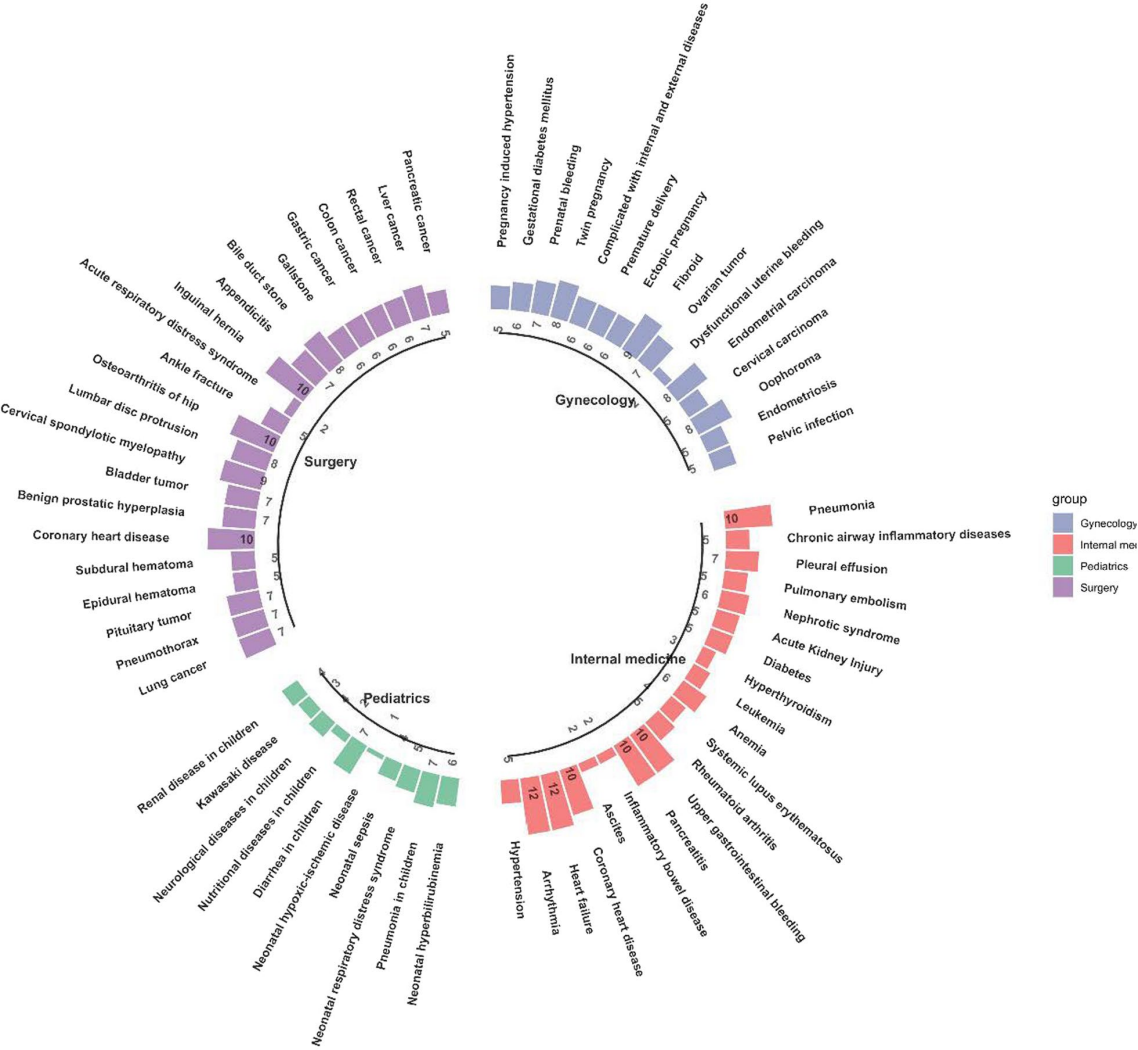
Proposed AIteach cases

Medical records for the proposed AIteach system were collected from the hospital data center (HDC) for a large hospital in China. The HDC adopted Hadoop big data architecture over the HDFS distributed file system, HBase columnar database, Hive data warehouse, and Mahout machine learning. Thus, it can easily provide data storage and analysis calculation [17], connect multi-source data, match rich intelligent application scenarios, and provide essential data foundations for virtual medical record teaching via human–computer interaction. Teacher select suitable cases for individual student learning and access complete real medical records, including 413 cases, covering 67 diseases, as shown in Fig. 1.

### Construction of virtual learning system AIteach

AIteach is mainly oriented to four groups of people: medical students (including undergraduates and graduate students), teachers, teaching administrators, and information administrators. The program has two user interfaces: (1) an AIteach App mobile application (2) a web-based application. In addition to the native Java language and Swift language, the application UI also adopts the cross-platform React.js language, which ensures that it can be used in iOS as well as Android systems. Such multi-platform compatibility serves to ensure that students have a consistent experience while learning simulated cases. Using the web application developed by the Vue CLI framework based on Vue.js helps to achieve rapid development, long-term update capabilities. The configured complete system is mainly used for user maintenance, permission control, real medical record data retrieval, virtual case review, revision and release, student learning behavior statistics, and other functions. Both front-end applications use the back-end application service based on the Spring Boot framework, realize the inversion of control through dependency injection to realize the containerization of the management object life cycle, and use a MYSQL database as the data storage of the application layer, which is a direct user-oriented behavior and operation to provide complete data access services. In addition, the NLP service of the big data service layer uses Python 3.7 as the core language. Using actual medical records, examination reports, and clinical literature guidelines stored in the HDC, it provides true and accurate information for the entire virtual case learning system.

### Virtual medical record generation

**Fig. 2:**
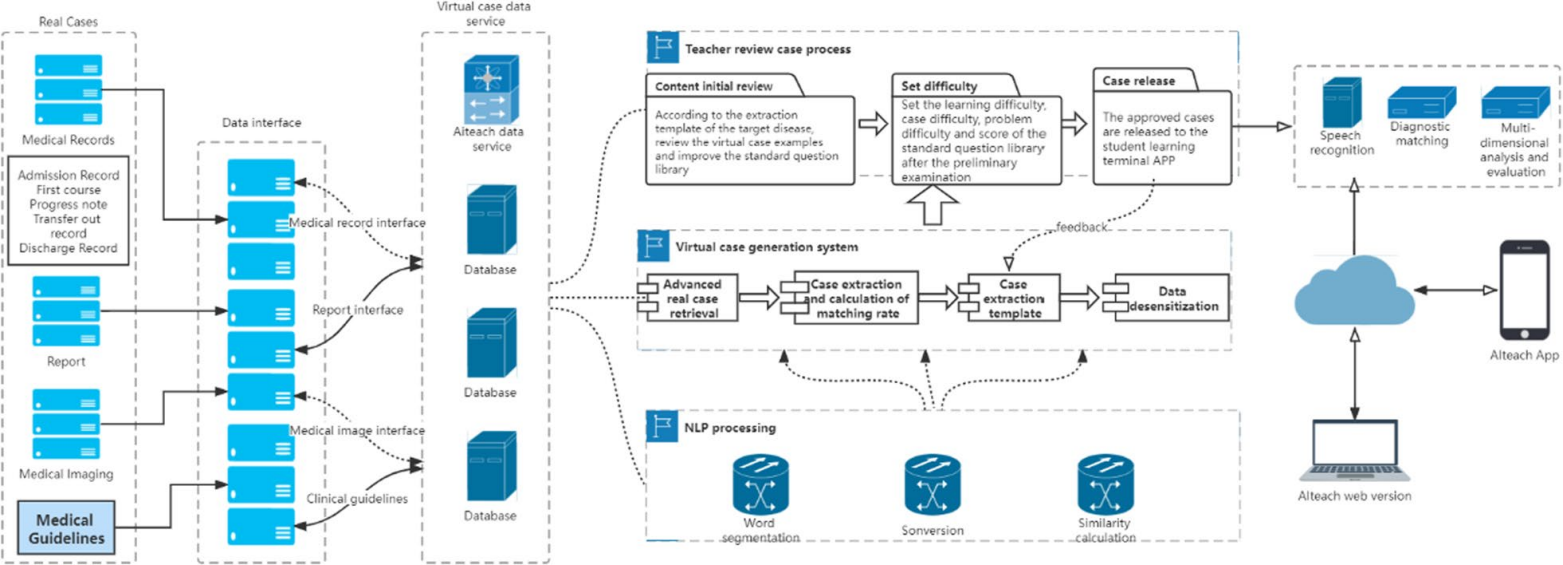
Virtual medical record generation process

AIteach was designed to interface with virtual patient simulator (VPS), a computer program that simulates real clinical scenes, enabling students to simulate doctor’s roles by obtaining medical history, conducting physical examination, and making diagnosis and treatment decisions [18, 19]. Figure 2 shows that generating virtual medical records comprises data extraction and medical record review.

#### Data extraction

**Fig. 3:**
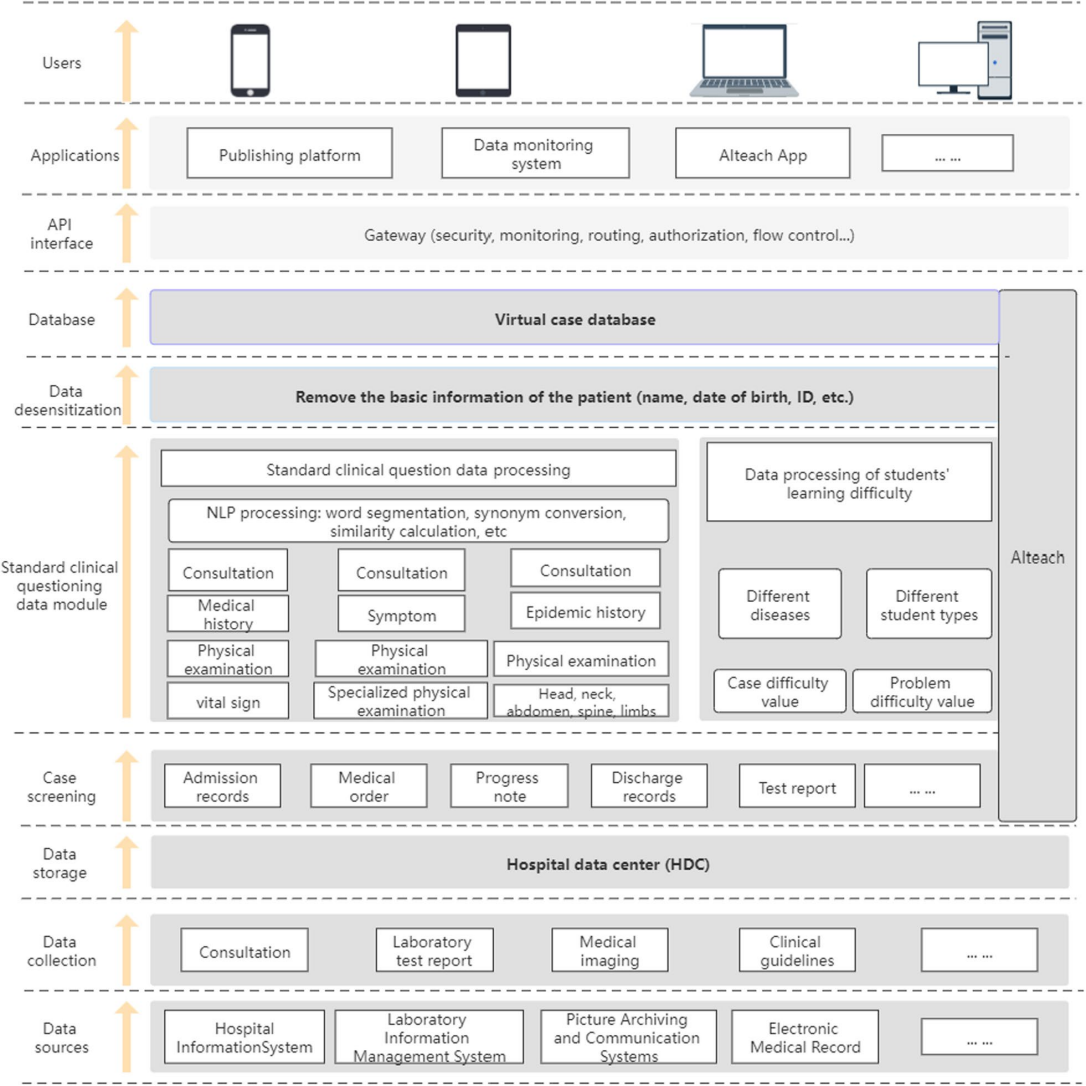
Proposed AIteach extraction process

Virtual cases were extracted from real cases, including structured and unstructured data. Structured data included test reports and basic patient information, and unstructured data comes from medical records. Figure 3 shows the virtual medical record extraction process.

- Data was acquired from various hospital information systems, including laboratory information management systems, picture archiving and communication systems, electronic medical records, etc.
- Data acquired in is stored in the HDC.
- Teachers retrieve and extract cases from the HDC depending on characteristics that meet desired clinical thinking training, such as diagnostic ICD code, symptoms, admission time, gender, and other retrieval conditions.
- Perform NLP on medical records from (c) and standard clinical questioning data processing through word segmentation, synonym conversion, similarity, etc., to obtain matching rate ranking for real and target cases.
- Submit cases with high matching rate (≥ 90%) in (d) to the review teacher who corrects final problem items based on actual case differences and then sets target student type and case difficulty to mark.
- Desensitize medical records that have undergone difficulty setting and standardized processing in (e). The Health Insurance Portability and Accountability Act (HIPAA) requires that personal privacy fields be desensitized by default, and the desensitization field can be customized. Irreversible basic patient information desensitization, such as name, ID number, and other important information, directly replace original information with the "*" symbol. Search and statistics using this sensitive information are not supported, and the real patient identity cannot be deduced from virtual medical records.
- Once the teacher has passed the review, virtual cases with privacy removed in (e) are stored in the virtual library as cases to be released. Students with case learning permissions can download corresponding released cases through the mobile phone app for study and clinical thinking training.
- Students can obtain final academic performance results after learning, track and analyze data in the learning process, and generate performance reports. They can also freely choose different learning difficulties for repeated training and learning.

#### Virtual medical record teacher review requirements

**Fig. 4:**
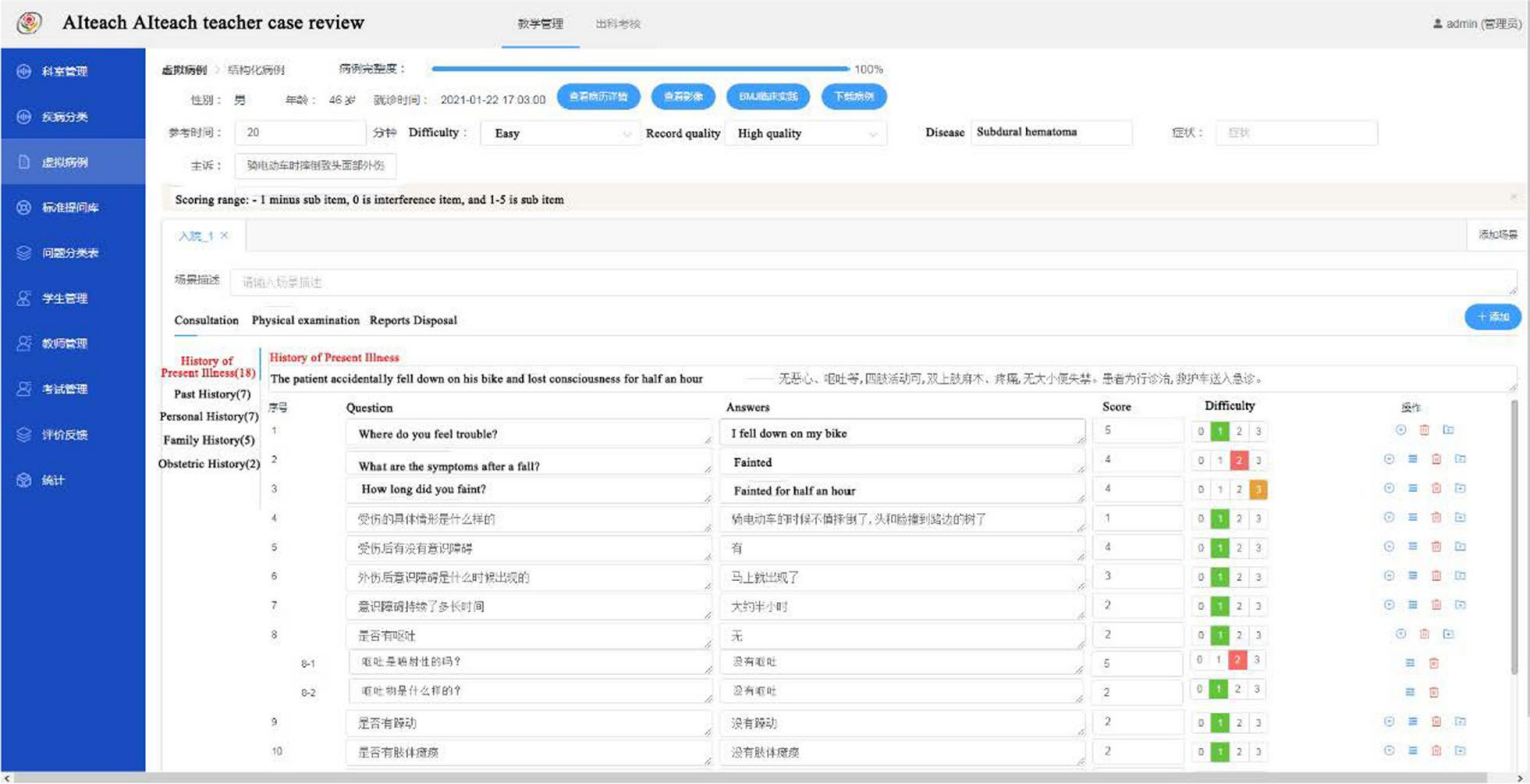
Proposed AIteach teacher case review interface

The basic construction form of a virtual case is “select questions-provide results-select questions-verify results”. Question design and thinking need to comply with the general principles of evaluation-selection-action-reevaluation [20]. The proposed AIteach system allows teachers to add screening conditions to real cases, identify real cases with moderate difficulty suitable for students to learn from, and use NLP to automatically extract virtual cases as questions and answers. Teachers are responsible for revising and reviewing case content, using the review tool shown in Fig. 4. It is particularly important to consider conceptual relationships between diagnostic factors and hypotheses, and whether it can guide students to the correct diagnosis [21].

Teachers who review AIteach cases must have more than ten years clinical practice experience and be responsible for daily teaching. They mark questions and answers for difficult virtual cases considering data mining of the diagnoses, treatment plans, and progression paths for different patients affected by the same disease, disease coverage, medical record typicality, and investigation content specificity. They can also add and delete question items to allow individualized customization and add specialized physical examinations into individual cases for various diseases and specialties. The complete process helps students cultivate critical and comprehensive clinical thinking.

### Natural language processing for case extraction

**Fig. 5:**
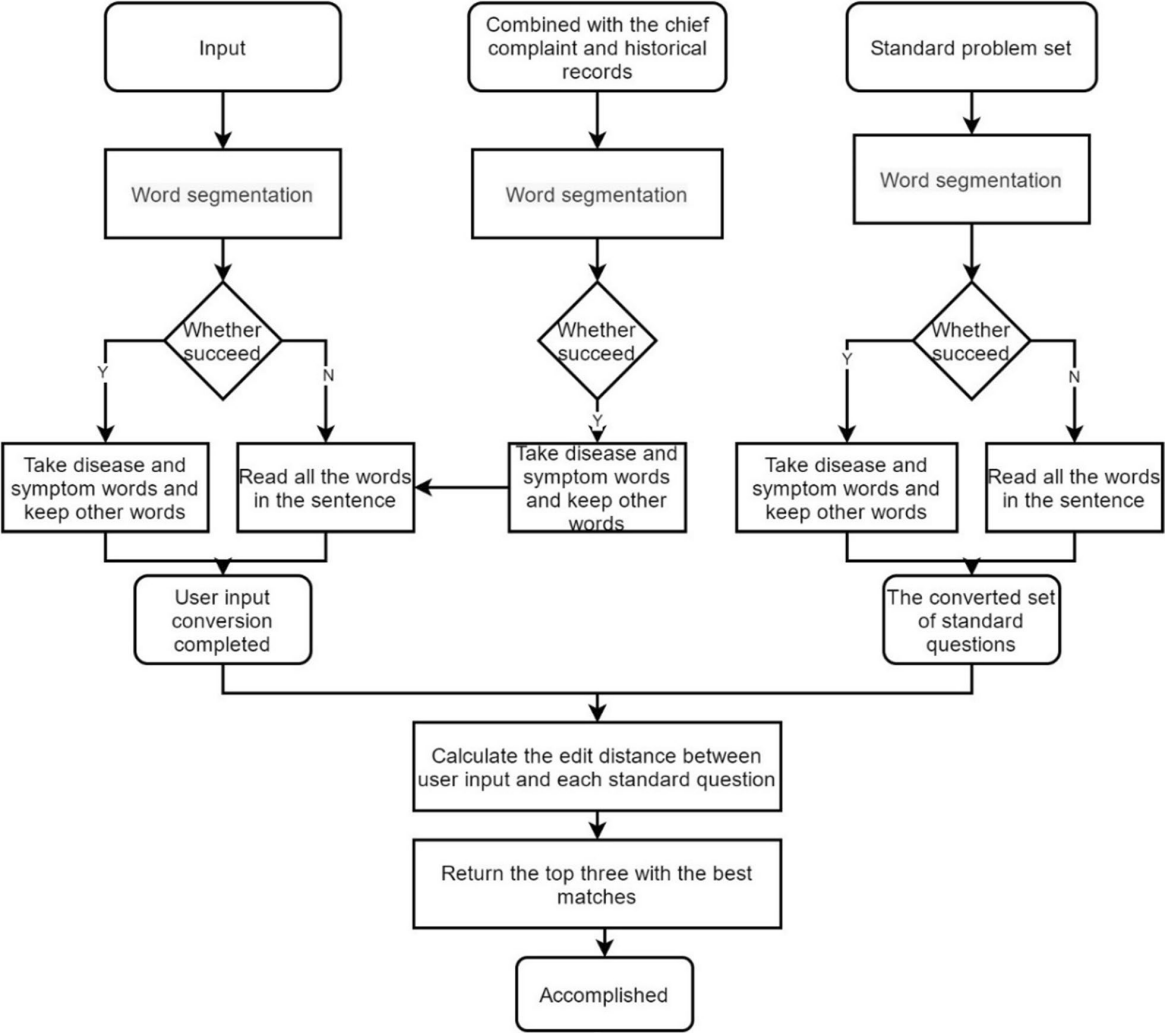
Natural language processing logic diagram

Virtual case teaching uses NLP to convert original cases into virtual question-and-answer cases and hence enable students to input questions and automatically match answers. The technical process involves word segmentation, synonym conversion, similarity calculation, and other processes to improve multi-round question-and-answer accuracy. The main complaint and other historical issues regarding medical records should also be added to question matching, as shown in Fig. 5. Given the large accumulated medical knowledge base, this solution uses a Chinese word segmentation algorithm based on the statistical language model, which employs Trie tree structures to realize word graph scanning [22], generating a directed acyclic graph [23] comprising all possible word formations from Chinese characters in a sentence. The system uses dynamic programming to find the largest probability path and determine the largest segmentation combination based on word frequency. We added a rule model to process numerical and template data commonly used in the medical field, broaden the scope of word segmentation scop, and add proper nouns to improve the accuracy of word segmentation.

Many common spoken words in the teaching process are often unknown words, hence the AIteach algorithm uses an Hidden Markov Model (HMM) model based on Chinese characters ability to form words and uses the Viterbi algorithm to enhance medical colloquial word segmentation accuracy. The HMM and other models were trained on 5000 medical record document data.

**Fig. 6:**
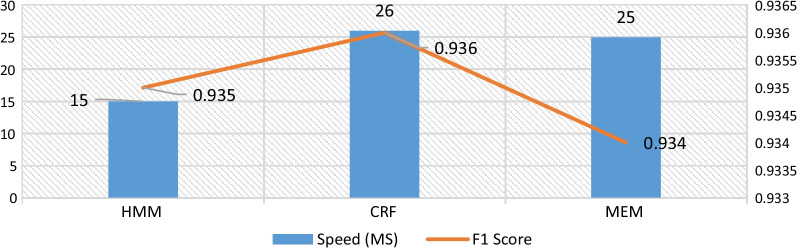
Performance metrics for HMM, (CRF), and MEM models on colloquial words

Figure 6 shows the achieved accuracy and efficiency for the test dataset. HMM achieves better performance than the other considered models. For example, the natural sentence “Morning hand stiffness yes or no?” in colloquial processing returns as “Are your hands stiff in the morning?” after Chinese word segmentation.

We used the word2vec conversion algorithm to convert the corpus into a vector model, converting words with similar semantics into vectors with similar values based on the vector space metric [24]. For example, the word “stiffness” in the above example is converted into a vector N using the pre-trained vector model ${\mathrm{W}}_{\uptheta }({\mathrm{w}}_{\mathrm{n}})={\uptheta }_{\mathrm{n}}$, and word vectors for all standard words are used to calculate similarity using cosine distance to obtain the nearest word vector “Morning stiffness”, whereas the other words remain unchanged.

The solution for negative words is to identify corresponding qualifiers, such as denial, negation, not excluding, without, not, not seen, none, etc. The above qualifiers are obtained by human experience in advance. Misspellings cannot be addressed automatically, and hence the teacher needs to recognize and correct misspellings when reviewing the medical records.

### Student input question and answer matching

Self-text clinical questions raised by students need to be converted into standard medical questions after word segmentation and synonym conversion. The converted questions and questions from the system standard question library are subsequently edited, and a score calculated to evaluate candidate question standard. If a standard question has zero edit distance from a student raised question raised, the result is returned as the student question. If 1 < distance < 5, it follows the specific order from small to big to give a set of candidate standard questions, then it asks the student to reconfirm the standard questions, and to give the corresponding answers to the questions according to the specific standard questions. If distance > 5, the student is informed there is no match, with the response “You have deviated from the correct process”.

### Cultivating students' clinical thinking with various difficulties

Students with various seniority have different requirements regarding knowledge acquisition and learning difficulty. The AIteach virtual case system meets these diverse needs by identifying case and learning difficulties. Case difficulty classification includes simple, moderately difficult, and complex cases. We established a four-level framework for learning difficulty to complete the diagnosis and treatment strategy.

- Level 1 provides students with a comprehensive version of the case and an answer reference to select from.
- Level 2 provides interference and error for history, physical, and auxiliary examinations.
- Level 3 requires students to enter more important scoring items themselves.
- Level 4 is fully independent self-study mode. Students need to enter all diagnosis and physical examination questions themselves.

Students can choose the specific difficulty level suitable for their present knowledge level and simulate disease progression in patients. Patient response to the treatment give students the opportunity to experience various aspects for any disease, and hence form overall impressions and improve their understanding.

## Results

### Virtual medical records

**Fig. 7:**
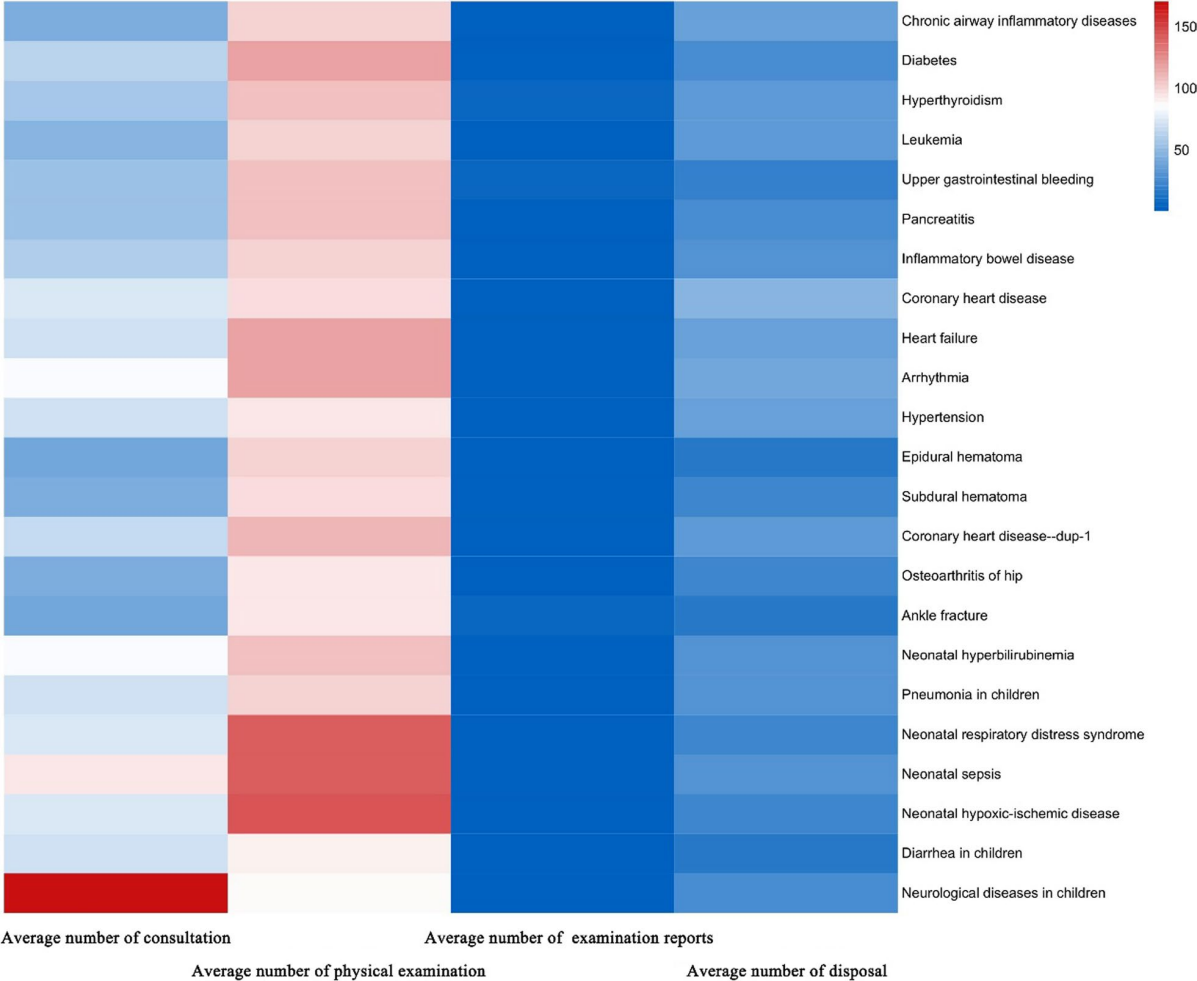
Information distribution for AIteach virtual cases

The AIteach virtual medical record database contains 413 cases, covering 67 diseases. Figure 7 shows average number of consultation questions, physical examinations, and reports. The number of physical examination questions exceeds that for other modules, and the largest number of questions are regarding pediatric cases.

### AIteach system double-cycle learning mode

**Fig. 8:**
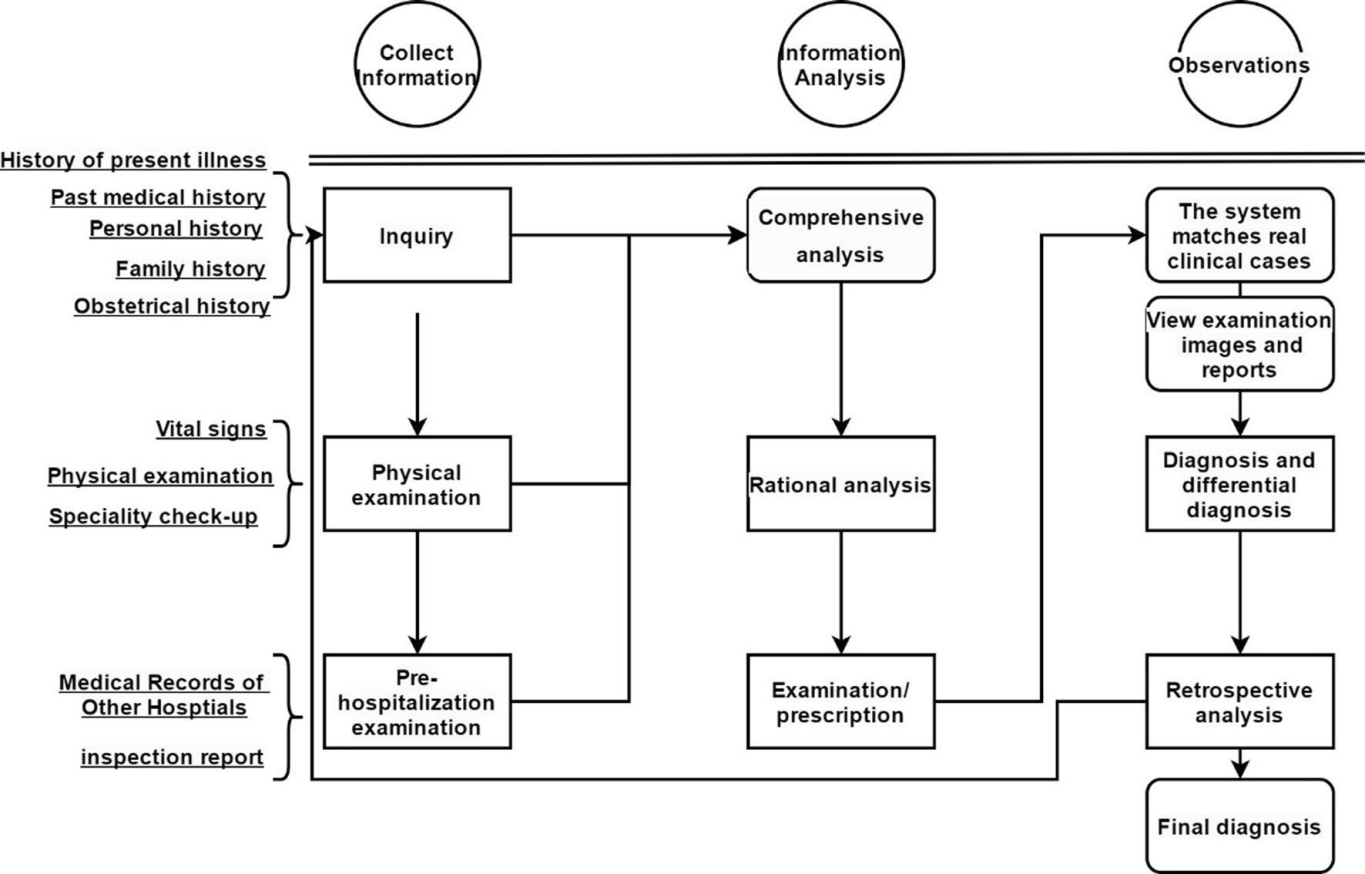
AIteach system’s double-cycle learning mode. The questioning scene is mainly to obtain medical history collection information, including history of present illness, past history, personal history, family history, and obstetric history. Physical examination includes: vital signs, general conditions (development, nutrition, face type, expression, and other aspects), skin and mucous membranes, lymph nodes, and other systems

Clinical thinking training should cultivate students’ ability to collect information, analyze information, and verify by observation; employing internal feedback based on gradual feedback and prompts while executing the learning [25] and an outer loop comprising analysis, observation, verification, collection, and analysis [26]. For the AIteach system, students first collect case information, analyze and consider the collects information, and finally observe and verify, as shown in Fig. 8. Following the inner loop feedback, each AIteach learning unit, e.g. Consultation, matches corresponding answers with inquiry questions. Students can review the questions at any time and add further questions. AIteach then matches student questions with standard questions and calculates the score.

**Fig. 9:**
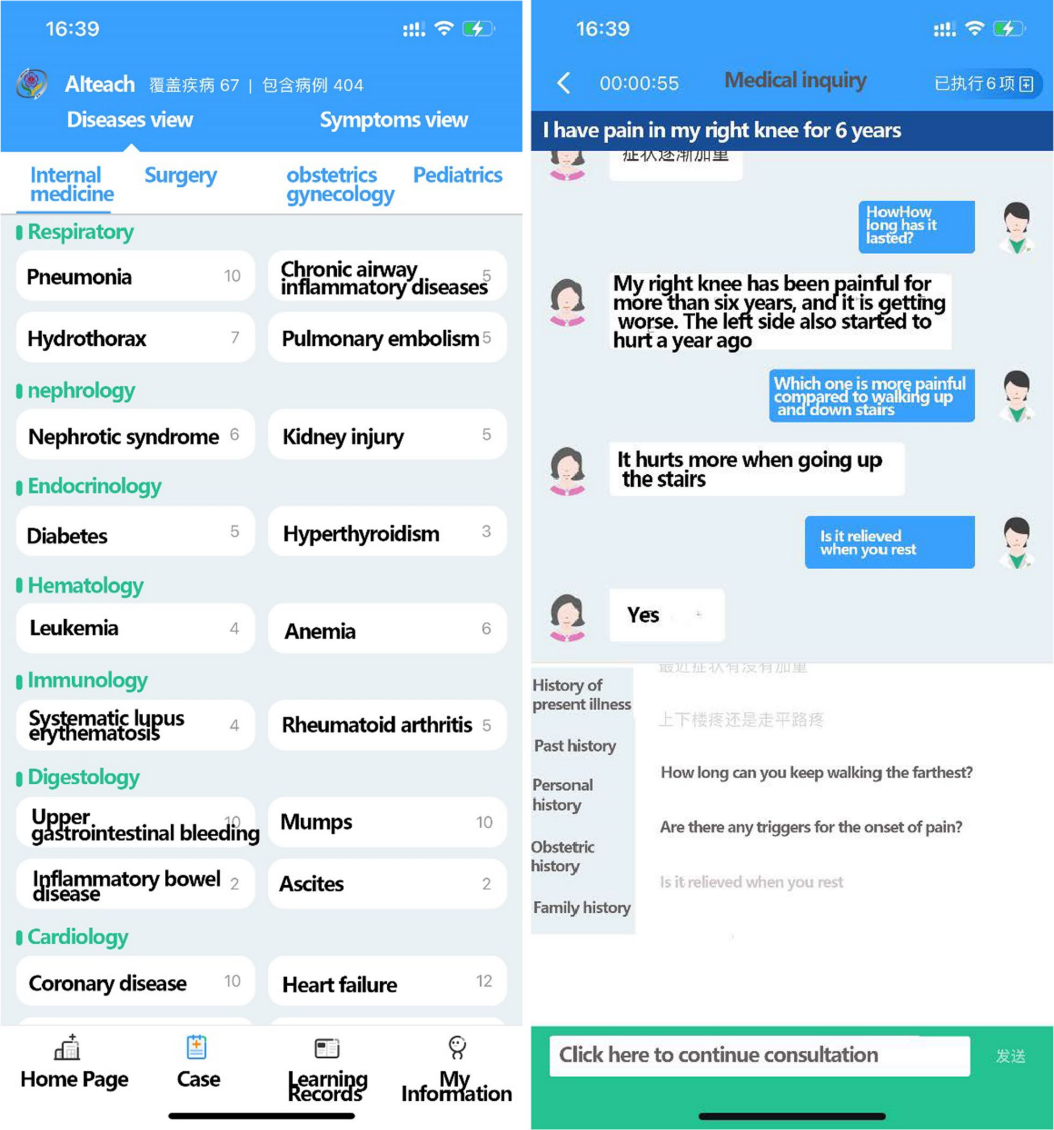
AIteach App system home page. Students can choose the learning cases on this interface (left picture). Students have virtual consultations in this scenario (right picture)

The AIteach system requires the student to re-select the diagnosis basis again after the initial condition description, analysis, diagnosis, differential diagnosis, and treatment plan, based on the questions and answers from the previous units. Students can visualize and weigh relationships between previously established diagnostic factors and assumptions using binary analysis. Figure 9 shows the proposed AIteach learning process.

Information collection includes interactive questions and answers related to consultation and examination in the medical scene. The process is precisely the same as dealing with patients in a clinical setting. AIteach also has specialized physical examinations, including gynecological, obstetric, and pediatric specialist examinations; which accurately reflect specific case characteristics for those disciplines.

AIteach allows students to switch between multiple medical treatment scenarios. It collects and analyzes supplementary information at any time based on information analysis. Students must learn to collect all diagnostic factors potentially related to the final diagnosis. This can only be achieved by students interacting in natural language and formulating their own questions and responses, rather than selecting questions from a predetermined list. Information analysis, based on recording student’s operation steps in the learning process, combining NLP algorithm mapping, and marking against the reference answer, allows students to develop diagnoses and differential diagnoses from the collected information, then the NLP algorithm processes the student’s diagnosis to determine correctness. The student's proposed diagnosis is considered correct if a matching disease appears in International Classification of Diseases Ontology reference list, and subsequently included in the student’s differential diagnosis. Once students believe their differential diagnosis is complete, they can analyze the data collection results (as shown in Table 1). AIteach automatically calculates and lists diagnostic factors and hypotheses determined by the students so far, and marks important missing items.

**Table 1:** Evaluation methods of clinical thinking ability in AIteach

Indicators	Calculation methods
Rigor of thinking	100 × (sum of the basic items under the correct diagnosis actually selected / sum of all the bases for correct diagnosis)
Logic of thinking	100 × (Number of selected − number of correct inquiry not selected + Number of selected physical examinations − number of correct physical examinations not selected)/ (correct number of inquiries + number of correct physical examinations)
Systematic thinking	100 × (total number of selected correct inquiries, physical examinations, diagnoses, diagnoses bases, and treatments)/ (total number of correct inquiries, physical examinations, diagnoses, diagnoses bases, and treatments)
Agility of thinking	100 × (4 × Recommended completion time /(4 × Recommended completion time + actual completion time)) × (Selected score / Total score)
Expansion of knowledge	100 × (Total score of selected diagnosis and diagnosis basis)/(Total score of diagnosis and diagnosis basis)

Medicine is a highly practical discipline, with considerable complexity and uncertainty regarding medical problems. Thus, student ability to properly use, analyze and judge all information types is very important, and critical thinking is essential for accurate medical diagnoses. AIteach designs virtual cases so that students first propose diagnosis and differential diagnosis, then use specific previous virtual interrogation, physical examination and auxiliary examination outcomes to select the subsequent diagnosis basis. This selection process helps students improve their critical thinking ability, allows a meaningful progress score, and provides feedback on this case after learning. Teachers can then view the feedback and guide students as appropriate.

### Multi-dimensional analysis and evaluation for clinical thinking ability

Clinical thinking ability involves extremely high requirements for the objectivity and standardization of evaluation. Hence, a multi-dimensional automatic evaluation system is required. Clinical thinking ability cannot be evaluated solely by scores or conclusions [27]. With reference to the residents standardized training report [28, 29], this study summarizes five major indicators (rigor of thinking, logic of thinking, systematic thinking, agility of thinking, and expansion of knowledge) to evaluate students’ learning and clinical thinking ability. Based on the scores of students in various dimensions of learning, a radar chart of clinical thinking ability is drawn to assess the compliance of medical students’ clinical thinking ability. Based on the total score, a quantitative assessment of students’ clinical thinking ability is provided. Among the indicators, rigor of thinking refers to the comprehensiveness of diagnosis and differential diagnosis; the more effectively the items are completed, the higher the score. Logic of thinking refers to the compliance of the logical order of consultation, physical examination, and diagnosis. Systematic thinking examines the correctness rate of students during the entire process. Agility of thinking refers to whether the time taken to respond is within the normal time range. The more effectively the items are completed and the shorter the time consumed, the higher the score. Expansion of knowledge investigates the accuracy of diagnosis and differential diagnosis, as well as whether the evidence of diagnosis and differential diagnosis is sufficient and the accuracy score is high.

### Short-term learning test results

**Fig. 10:**
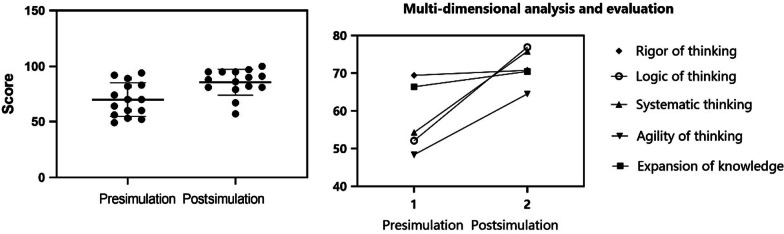
Overall performance changes of all students before and after the virtual case study. After using AIteach (left picture), the average value of the overall case simulation training evaluation has been significantly improved. It also significantly improved under the multi-dimensional index evaluation (right picture)

**Fig. 11:**
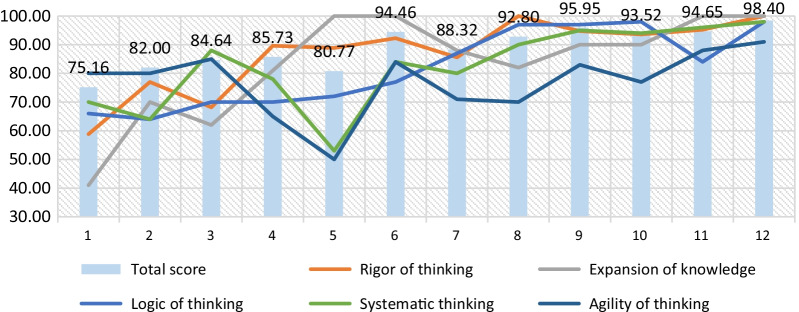
One student’s performance changes before and after learning multiple virtual cases

Fifteen students participated in our experiment for improving clinical thinking using virtual cases. The medical records were of identical difficulty; the learning difficulty was level 2 difficulty with interference items. The full score was 100 points. The results before the virtual case study were compared with the results after case simulation performed in AIteach. The average training evaluation score was observed to have improved significantly. The average before learning is 69.87 with a standard deviation of 14.69, whereas the average after learning is 85.6 with a standard deviation of 11.31, *P* < 0.01, as shown in Fig. 10. The individual performance of the students is shown in Fig. 11. It can be clearly observed that all students have improved, with different individual degrees of improvement. According to the analysis of five latitude indicators, there is a significant improvement before and after using the AIteach system, with logical thinking improvement being the most obvious (47%). After a student has studied 12 times, his grades have gradually improved, as shown in Fig. 11.

## Discussion

### Comparison of virtual case study method and traditional clinical thinking teaching method

The assessment and evaluation of clinical thinking ability of medical students and resident physicians is a challenging task. At present, commonly used clinical thinking evaluation methods include case analysis [30], project-based learning [31], teaching rounds [32] mini-CEX [33, 34], and standardized patients [35, 36]. These common methods are restricted by many factors, such as location restrictions. In order to avoid leaks, the multi-station assessment of clinical thinking ability often requires multiple classrooms, and each examinee needs to take the test in a fixed examination room within a fixed time limit. Invigilator teachers and volunteers conduct assessments and work at a uniform time. Pre-test training, test guidance, on-site scoring, post-test scores, and examination room layout and arrangement take up more time. Environmental constraints, medical students, and resident physicians are independent entities. There are fewer opportunities for diagnosis and treatment, or even communication with patients privately. Furthermore, many patients do not understand and support learning and training work; when medical students ask for medical history, some patients will not truthfully answer questions, especially regarding personal information such as smoking habits. Organizing a multi-site assessment of clinical thinking ability requires considerable manpower, including diagnostics and clinical teaching experts, teachers, and volunteers. This study issued questionnaires to 15 students and evaluated the comprehensiveness of the virtual medical cases, the accuracy of information analysis to guide diagnosis, the efficiency of the students’ study, and the system usability. Fifteen questions were presented. The results are shown in Table 2. AIteach provides medical students with rich, safe, and convenient virtual clinical practice opportunities, organically integrates online learning, practice error correction, feedback evaluation, and other teaching processes, and builds a link between independent learning and clinical practice. The AIteach virtual case system can further expand clinical teaching resources and overcome the lacunae of relatively insufficient and non-repeatable case resources in the clinical practice process as well as multiple progression paths in disease conditions [37].

**Table 2:** Students’ feedback and evaluation of virtual medical record teaching method

Number	Area	Question	Very satisfied	Satisfied	Generally satisfied	Dissatisfied	Very dissatisfied	Total number of students
1	The comprehensiveness of virtual medical cases	The comprehensiveness of history of present illness	9	4	2	0	0	15
2		The comprehensiveness of past history	8	4	3	0	0	15
3		The comprehensiveness of personal history	7	5	3	0	0	15
4		The comprehensiveness of family history	10	3	2	0	0	15
5		The comprehensiveness of obstetric history	10	4	1	0	0	15
6		The comprehensiveness of examination reports	7	7	0	0	0	15
7	The accuracy of information analysis to guide diagnosis	Guide to infer the correctness of diagnosis from the examination reports	8	5	2	0	0	15
8		Guide to infer the correctness of diagnosis from the inquiry	10	4	1	0	0	15
9	Efficiency of students’ study	Master the skills of inquiry and diagnosis of the disease	10	3	2	0	0	15
10		The physical examination methods of the disease were mastered comprehensively and orderly	9	3	3	0	0	15
11		Learn how to deal with the disease	7	4	4	0	0	15
12	System usability	Short download and installation time	10	4	0	1	0	15
13		High fluency when loading and converting interface	9	5	0	1	0	15
14		High stability	12	3	0	0	0	15
15		The operational design of the button is user-friendly	14	1	0	0	0	15

Students reported that the learning mode of virtual medical record is comprehensive, full guidance, effective for learning, and easy to use

### Cultivation of clinical thinking covering the entire scenario

The AIteach system collects and analyzes all-scene information, including medical inquiry, physical examination, and laboratory examination. Medical students must make decisions in each step, and each decision is a manifestation of clinical thinking ability. Each decision ultimately constitutes a decision sequence. Students arrive at different decisions each time and get different multi-dimensional evaluations. Each decision requires medical students to think independently, actively participate, and finally form conclusions such as the diagnosis and treatment. The AIteach system helps medical students establish overall clinical thinking and disease analysis awareness. Using mobile phones, students can repeatedly study cases anytime and anywhere, independently ask questions, analyze problems, solve them, learn the etiology, pathogenesis, symptomology, health education and other characteristics of the disease, accumulate clinical experience, and train their clinical thinking ability. Especially for rare and complex diseases, AIteach can effectively compensate for the disconnect between basic theoretical knowledge and actual clinical work [38].

### Assist teachers directly to obtain teaching information

The system automatically records the student’s erroneous diagnoses and feedback to the teacher. As presented in Table 3, the teacher can specifically teach the erroneous diagnosis in detail, providing a platform for teachers to continuously develop and refine clinical teaching goals.

**Table 3:** Correct and wrong diagnosis records of students’ learning automatically recorded by the system

Virtual case ID	Disciplines	Disease	Overview	Learning times	Diagnosis	Name	Types of students	Correct diagnosis selected	Error diagnosis selected
becb6c06-e6ba-41d3-96bc-7dbb7a8fafdb	Gastroenterology dept	upper gastrointestinal hemorrhage	Black stool for 10 days	5	Chronic gastritis, Duodenal ulcer	A	Postgraduate	Chronic gastritis, Duodenal ulcer	None
9ec05367-c942-4a9e-a024-6577f1338341	General surgery department	Inguinal hernia	A mass in the right groin area was found for 2 years and pain for half a year	1	Right inguinal hernia	B	Undergraduate	Right inguinal hernia	Lipoma in the groin area

### Limitations

The process of interviewing a patient involves face-to-face communication, a physical examination during the process of asking the medical history, identifying issues during the examination, and then supplementing the consultation. There are few clinical colloquial corpus in NLP field. This affects the preparation of oral recognition matching. Owing to the limitations of NLP, although the teaching cases in this system are extracted from real medical records, they are released after removing privacy-infringing information and after a manual review. Therefore, there are certain differences from actual clinical practice.

Students should also provide evaluation feedback after learning virtual cases, and their improved critical thinking should make this feedback timely and relevant. Future study will consider adding timely feedback and guidance directly from doctors in the learning process.

## Conclusion

The shortage of human resources, the increasing cost of education, and the need to maintain social distancing in response to the global outbreak of the COVID-19 have all contributed to the need for clinical thinking training methods designed for distance learning. Through the intelligent virtual case-learning method of the AIteach system, each medical student can independently develop basic clinical thinking skills such as making inquiries, conducting physical examinations, and disposal according to difficulty levels. Thus, they can learn from real cases with zero risk and high efficiency. The system can also provide multi-dimensional evaluations that address deficiencies in the learning process. The AI-based teaching method fully considers students’ clinical level and learning requirements. This kind of clinical thinking ability training mode not only has a sense of clinical reality, but also summarizes the common characteristics of common diseases, which substantially improves the quality and efficiency of teaching. AIteach is not a substitute for actual clinical practice; rather, it complements it by enabling teaching hospitals to make full use of their collection of big data related to medical cases. A new and enhanced paradigm for teaching concepts can be developed for medical students to increase their knowledge, improve clinical skills and clinical thinking ability, and grow into qualified clinicians.

## Data Availability

The data that support the findings of this study are available from the corresponding author upon reasonable request.

## Abbreviations

NLP: Natural Language Processing
VPS: Virtual Patient System
HDC: Hospital Data Center
HMM: Hidden Markov Model
